# Pilomatricoma: A Case Report

**DOI:** 10.7759/cureus.63574

**Published:** 2024-07-01

**Authors:** Sunanda Devi Putta, Rashmi P Rajashekhar, Tanmay Chaudhari, Gundappa Mahajan

**Affiliations:** 1 Otolaryngology, Head and Neck Surgery, Dr. D. Y. Patil Medical College, Hospital and Research Centre, Pune, Pune, IND

**Keywords:** histopathology, pilomatricoma, external ear, swelling, benign

## Abstract

Pilomatricoma is one of the rare skin benign neoplasms arising from the matrix cells of hair follicles. We report the case of a 17-year-old female who had a swelling over the medial surface of the right ear lobule, which was determined to be an epidermal inclusion cyst by radiological and clinical examination. Over the period of eight days, it grew to 2 × 2 cm. A well-defined encapsulated lesion with thick cellular debris was found on an ultrasonography of the local region. Under local anesthesia, the patient had a cyst excision. Early detection by ultrasonography is beneficial in skin lesions to confirm if they are benign or malignant. Complete excision of the cyst is the treatment in order to limit the morbidity and lower the aggressive behavior. In this case, we came to a diagnosis after a histopathological examination, confirming it as a pilomatricoma. Due to its rarity, it is often misdiagnosed.

## Introduction

Pilomatricomas are skin-benign neoplasms arising from follicles of the hair, with an approximate incidence of 1% among benign skin lesions. They appear as firm nodules, over half of the lesions where calcium deposits are identified. Basaloid and ghost cells are present in the island of epithelial cells, which are classically present [1]. The lesions are derived from the sebaceous gland. Other benign lesions like keratoacanthoma, ossifying hematoma, and fibroxanthoma are all subcutaneous nodules that should be differentiated from pilomatricoma [1].

Pilomatricomas are also known as calcifying epithelioma of Malherbe, as they were first described by Malherbe and Chenantais [1]. They are painless, subcutaneous tumors and lesions fixed to overlying skin but mobile about deep planes. A bluish ulceration or discoloration can be seen on the overlying skin [2].

These are more common in females. The female-to-male ratio is 2:1, and they are most commonly seen in the head and neck region, followed by the periorbital and preauricular region [3].

## Case presentation

**Figure 1:**
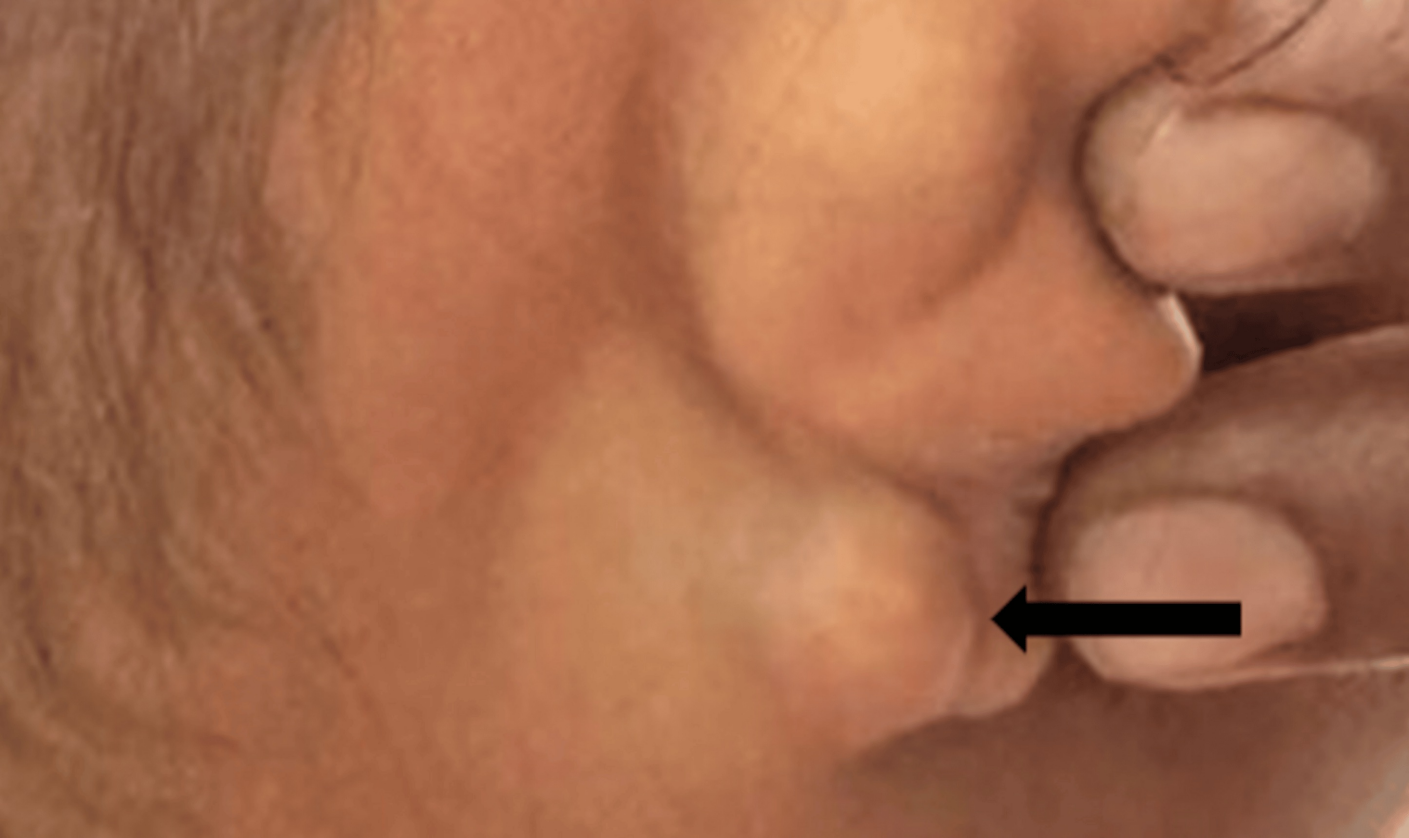
Swelling present over the medial surface of the right ear lobule (black arrow).

A 17-year-old female presented to OPD with swelling over the medial surface of the right ear lobule for 18 days (Figure 1).

It was insidious in onset and gradually progressive. There was no history of pain over the swelling or any other similar swelling present anywhere on the body. There was no history of any piercing over the local region or any associated discharge. Family history was not significant.

On inspection, 2 × 2 cm solitary swelling was present over the inner aspect of the right lobule. The skin over the swelling was normal. The swelling was without discharging pus. On palpation, all inspection findings were confirmed. The swelling was firm to hard in consistency, non-tender, and without the local rise of temperature. The skin over the swelling was fixed. The external auditory canal and the tympanic membrane were normal bilaterally. The other ear lobule and pinna were normal. The rest of the otolaryngology and head and neck examinations were normal.

**Figure 2:**
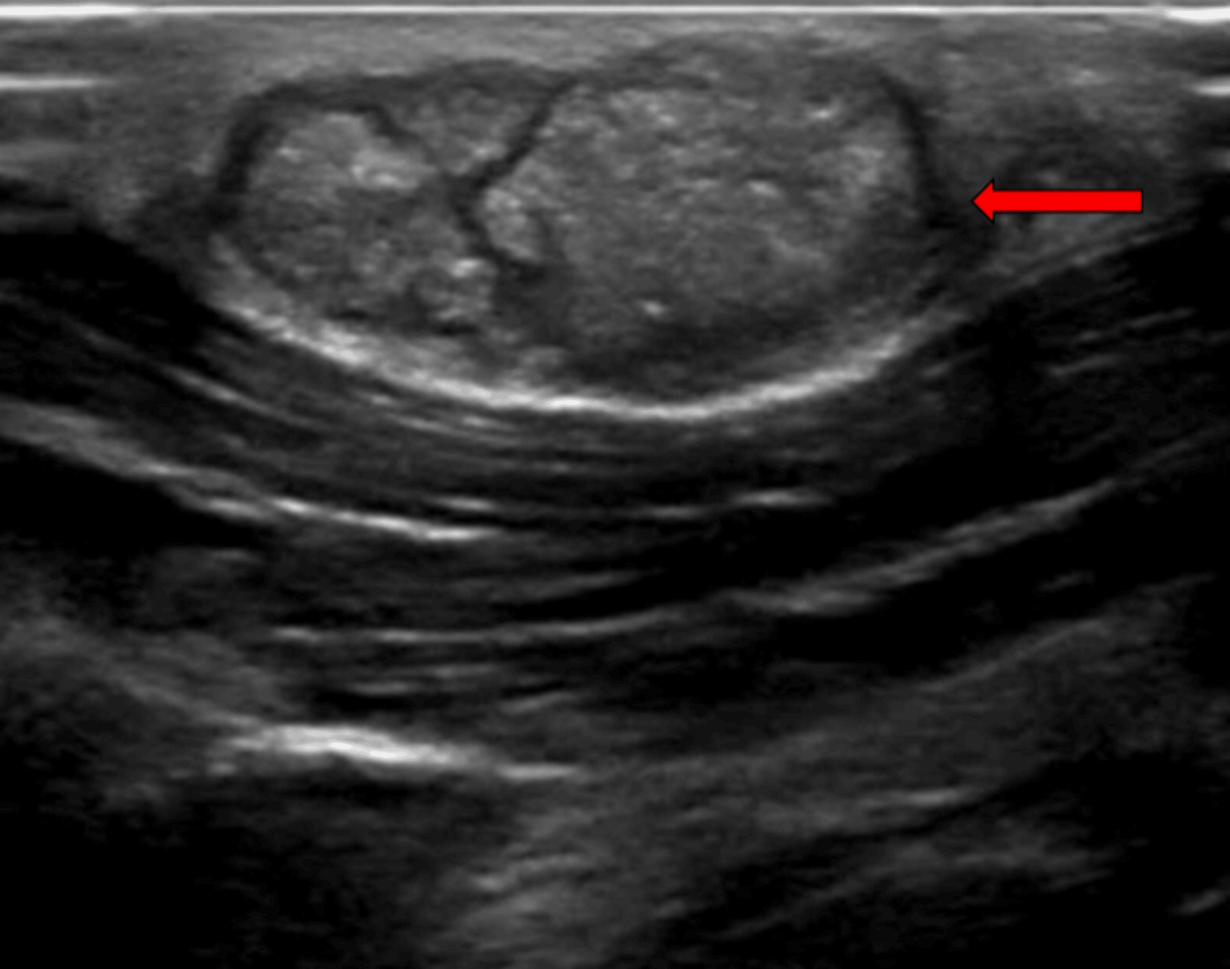
Ultrasonography of the local region (red arrow) showing a single well-defined encapsulated lesion with proteinaceous material within the subcutaneous plane.

All routine blood investigations were within normal limits. Ultrasonography of the local region showed a single well-defined encapsulated lesion with thick cellular debris or proteinaceous material within the subcutaneous plane in the right postauricular region. The following features were suggestive of a benign lesion, most likely an epidermal inclusion cyst (Figure 2).

After obtaining fitness for surgery from an anesthesiologist and written informed consent from the patients and relatives, the patient was taken up for the excision of an epidermal inclusion cyst under local anesthesia. The swelling was excised, hemostasis was achieved, and the procedural and postoperative periods were uneventful.

**Figure 3:**
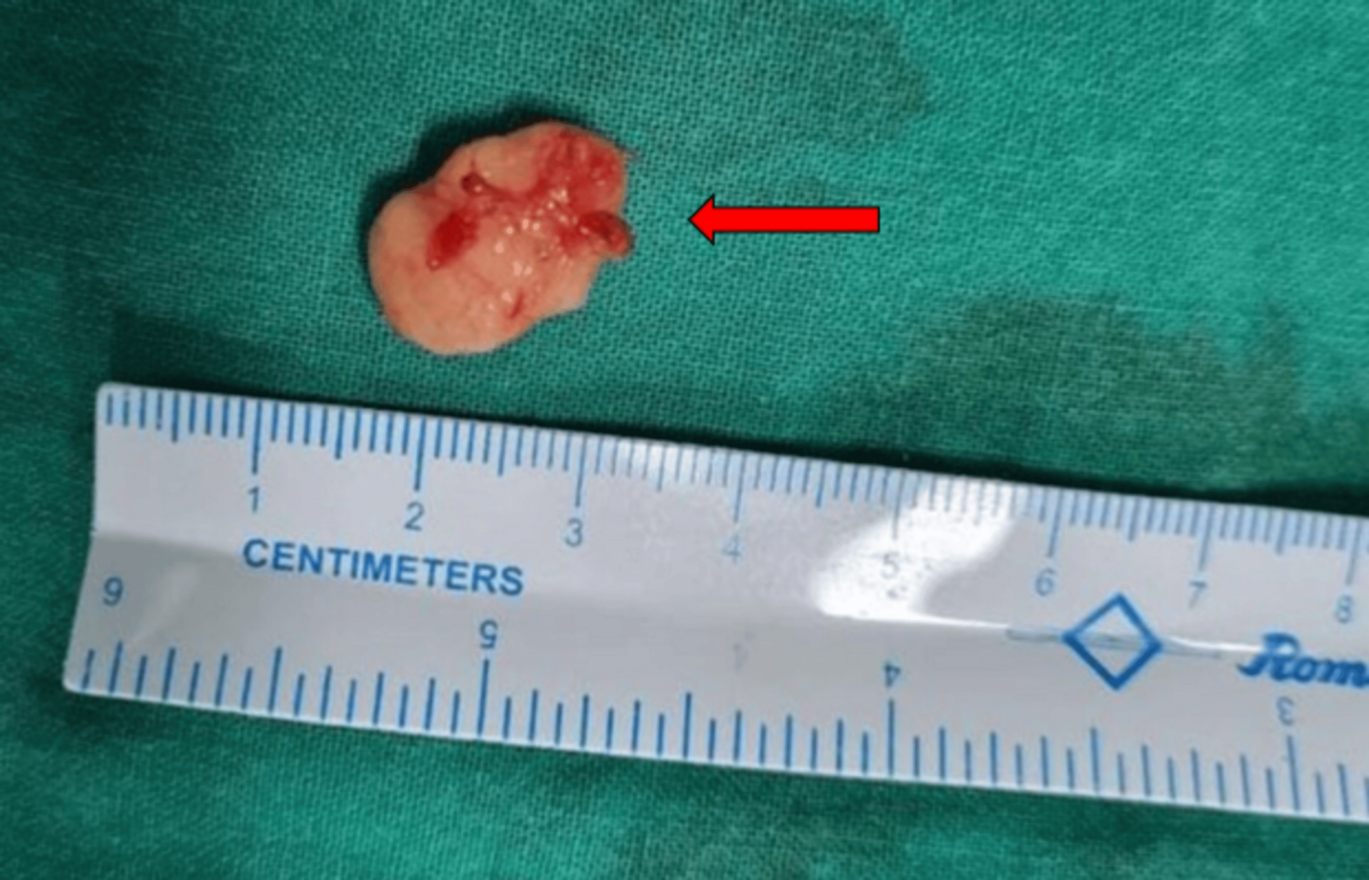
Firm, irregular mass of size 1.5 × 1 cm, excised from the medial aspect of right ear lobule (red arrow).

Further, the excised mass (Figure 3) was sent for histopathological testing.

**Figure 4:**
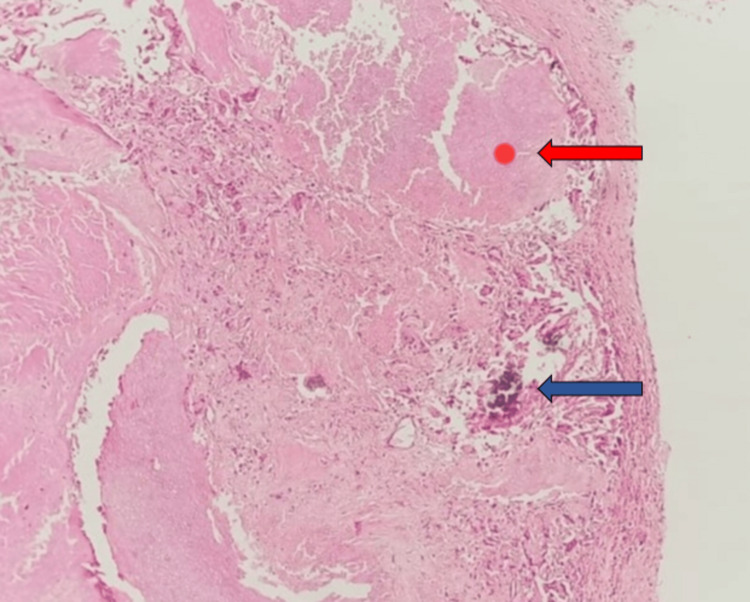
Histopathological examination showing a well-circumscribed tumor composed of islands of basaloid cells along with many ghost cells. Red arrow shows ghost cells and basaloid cells; blue arrow shows calcification.

Specimen for histopathological examination (HPE) (Figure 4) showed a skin-covered tissue showing epidermis.

The dermis showed a well-circumscribed tumor composed of islands of basaloid cells exhibiting abrupt keratinization without intervening granular layer along with many ghost cells. Sparse mitotic activity was noted. Intermediary cells showed eosinophilic cytoplasm with pyknotic nuclei. Lamellated flakes of keratin along with moderate mixed inflammatory infiltrate and numerous giant cells were seen.

The patient had a very fast recovery. She had follow-ups after one week, one month, and three months. No recurrence, gaping, or swelling was seen.

## Discussion

Pilomatricoma is a subepidermal tumor, which arises from proto-epithelial cells. Normally, symptoms like a small, hard lump under the skin are present. Two signs are seen in pilomatrixoma: one is the tent sign, where stretched skin is seen over the calcification of tumors to form a tent-like structure, and the second one is the teeter-totter sign, where pressing the mass from one edge causes the opposite edge to just out from the skin [4].

Pilomatricoma is a condition associated with some syndromes like Gardner's syndrome, Rubinstein-Taybi syndrome, gliomatosis cerebri, and also Steinert's disease (an autosomal dominant condition characterized by cataract, hypotonia, muscle wasting, and progressive mental retardation) [5]. Mutation in beta-catenin is frequently associated with pilomatricoma. It contains a 92kDa protein, which regulates function and normal cell growth. Some studies have shown that CTNNB1 gene mutation encodes beta-catenin, which is a cadherin protein component. Signaling in the Wnt pathway affects multiple cellular biological processes [6].

Morphological stages are present, which are four in number: 1) cystic, small lesions, which are the early stage; 2) cystic, large lesions, which are fully developed; 3) shadow cells and basaloid cells in foci, early regressive; 4) basaloid cells absent, shadow cells predominant, late regressive stage [7]. This condition mimics various other conditions like trichilemmal cysts, dermoid and epidermoid cysts, cutaneous T-cell lymphoma, folliculitis, dermatological manifestations of neurilemmoma, and metastatic carcinomas. The transformation from pilomatrixoma to pilomatrix carcinoma is rare, manifesting as non-tender, firm lesions resembling pilomatrixoma. There is a condition known as aggressive pilomatrixoma, where some studies suggest pilomatrixoma proliferating type to be a precursor of pilomatrix carcinoma [8]. Dermoscopy is helpful in pilomatricoma, showing bluish areas and white streaks, homogenous white areas, linear and dotted vessels, and erythema.

The position, degree of calcification, and the lesion continuity of the structures, which were deeper, can be demonstrated by pilomatricoma. Ultrasonography, CT, and MRI were also used in the phase of diagnosis. Pilomatricoma diagnosis can be missed with needle aspiration or through small biopsies [9].

Wide resection is recommended to minimize the risk. Most recurrences arise from incompletely excised neoplasms. Surgical excision is mandatory [10]. In 15% of cases, osseous metaplasia is seen [10]. A very small number of this condition can become cancerous. Recurrence rate is also present.

## Conclusions

In conclusion, this case highlighted the need to determine the swelling type following a comprehensive clinical history and histopathological assessment. A benign tumor called a pilomatricoma, which originates from a hair matrix, typically appears during the first 20 years of life. Seldom is this regarded as a differential diagnosis for benign masses, the identity of which is mostly determined by an HPE of the removed material. In most cases, surgical excision serves as both a diagnostic and a therapeutic measure.
